# Supplementary material for: Novel Driver Strength Index highlights important cancer genes in TCGA PanCanAtlas patients
Source: PeerJ. 2022 Aug 11;10:e13860. doi: 10.7717/peerj.13860 (PMC9375969; doi:10.7717/peerj.13860)
Supplement: Supplemental Information 2 [file peerj-10-13860-s002.zip › COHORTS/genes plots/age/2021_8_16_14_9_distribution_cohorts_males__25.pdf]

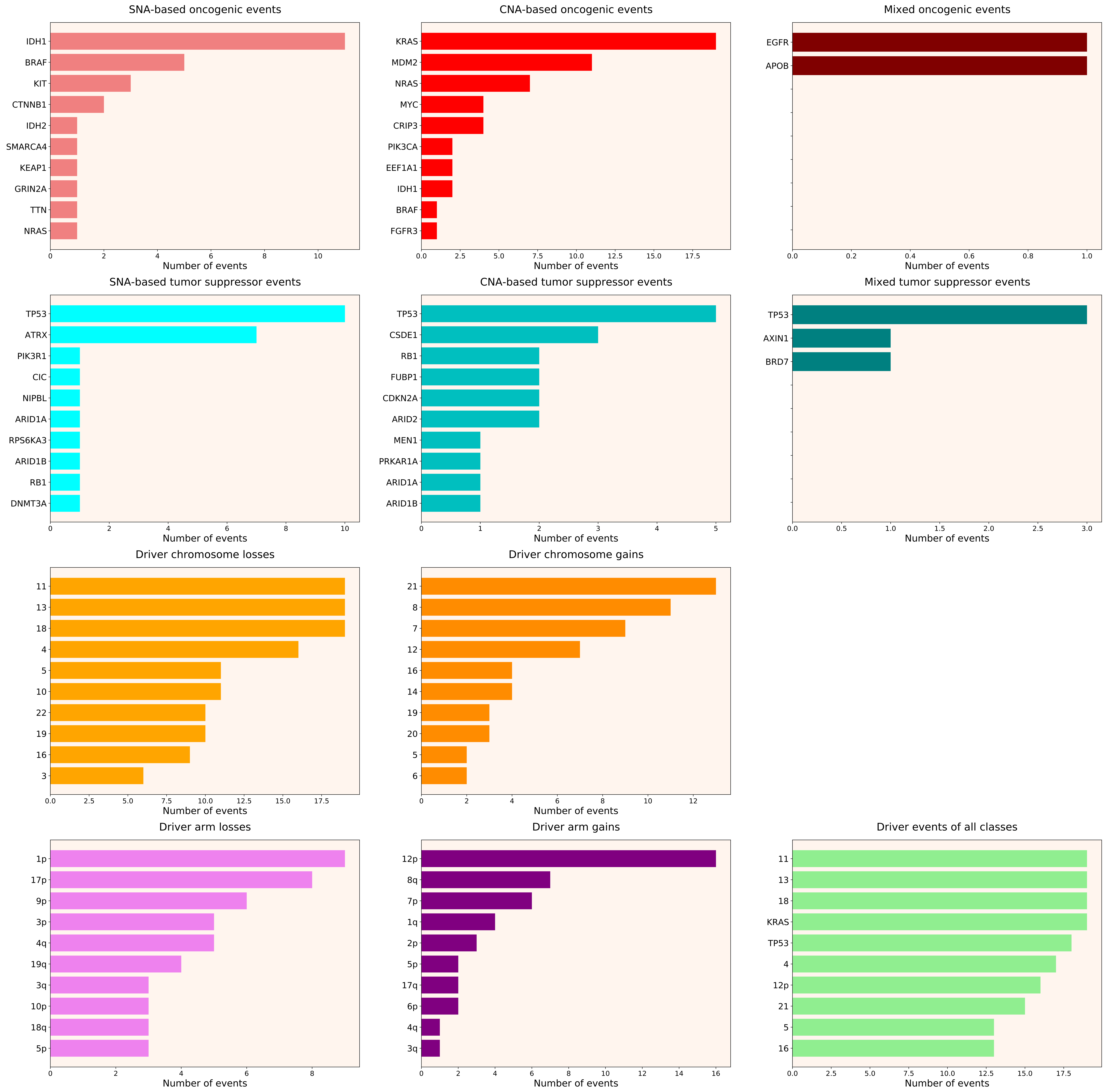

SNA-based oncogenic events
IDH1
BRAF
KIT
CTNNB1
IDH2
SMARCA4
KEAP1
GRIN2A
TTN
NRAS
0 2 4 6 8 10
Number of events
CNA-based oncogenic events
KRAS
MDM2
NRAS
MYC
CRIP3
PIK3CA
EEF1A1
IDH1
BRAF
FGFR3
0.0 2.5 5.0 7.5 10.0 12.5 15.0 17.5
Number of events
Mixed oncogenic events
EGFR
APOB
0.0 0.2 0.4 0.6 0.8 1.0
Number of events
SNA-based tumor suppressor events
TP53
ATRX
PIK3R1
CIC
NIPBL
ARID1A
RPS6KA3
ARID1B
RB1
DNMT3A
0 2 4 6 8 10
Number of events
CNA-based tumor suppressor events
TP53
CSDE1
RB1
FUBP1
CDKN2A
ARID2
MEN1
PRKAR1A
ARID1A
ARID1B
0 1 2 3 4 5
Number of events
Mixed tumor suppressor events
TP53
AXIN1
BRD7
0.0 0.5 1.0 1.5 2.0 2.5 3.0
Number of events
Driver chromosome losses
11
13
18
4
5
10
22
19
16
3
0.0 2.5 5.0 7.5 10.0 12.5 15.0 17.5
Number of events
Driver chromosome gains
21
8
7
12
16
14
19
20
5
6
0 2 4 6 8 10 12
Number of events
Driver arm losses
1p
17p
9p
3p
4q
19q
3q
10p
18q
5p
0 2 4 6 8
Number of events
Driver arm gains
12p
8q
7p
1q
2p
5p
17q
6p
4q
3q
0 2 4 6 8 10 12 14 16
Number of events
Driver events of all classes
11
13
18
KRAS
TP53
4
12p
21
5
16
0.0 2.5 5.0 7.5 10.0 12.5 15.0 17.5
Number of events
